# Risk factors and outcomes of organ-space surgical site infections after elective colon and rectal surgery

**DOI:** 10.1186/s13756-017-0198-8

**Published:** 2017-04-21

**Authors:** Aina Gomila, Jordi Carratalà, Daniel Camprubí, Evelyn Shaw, Josep Mª Badia, Antoni Cruz, Francesc Aguilar, Carmen Nicolás, Anna Marrón, Laura Mora, Rafel Perez, Lydia Martin, Rosa Vázquez, Ana Felisa Lopez, Enric Limón, Francesc Gudiol, Miquel Pujol, on behalf of VINCat colon surgery group

**Affiliations:** 1 Hospital Universitari de Bellvitge-IDIBELL, Barcelona, Spain; 2 VINCat program, Catalonia, Spain; 3 University of Barcelona, Barcelona, Spain; 4 Hospital General de Granollers, Universitat Internacional de Catalunya, Barcelona, Spain; 5 Parc Sanitari Sant Joan de Déu de Sant Boi, Barcelona, Spain; 6 Consorci Sanitari de Terrassa, Barcelona, Spain; 7 Hospital Universitari Mútua de Terrassa, Barcelona, Spain; 8 Consorci Sanitari de l’Anoia, Barcelona, Spain; 9 Corporació Sanitària Parc Taulí, Barcelona, Spain; 10 Fundació Althaia, Barcelona, Spain; 11 Hospital de Viladecans, Barcelona, Spain; 12 Hospital Universitari Sant Joan de Reus, Tarragona, Spain; 13 Infectious Diseases Department, Hospital Universitari de Bellvitge, Feixa Llarga s/n, 08907 L’Hospitalet de Llobregat, Barcelona, Spain

**Keywords:** Surgical site infections, Organ-space surgical site infections, Colorectal surgery, Surveillance

## Abstract

**Background:**

Organ-space surgical site infections (SSI) are the most serious and costly infections after colorectal surgery. Most previous studies of risk factors for SSI have analysed colon and rectal procedures together. The aim of the study was to determine whether colon and rectal procedures have different risk factors and outcomes for organ-space SSI.

**Methods:**

A multicentre observational prospective cohort study of adults undergoing elective colon and rectal procedures at 10 Spanish hospitals from 2011 to 2014. Patients were followed up until 30 days post-surgery. Surgical site infection was defined according to the Centers for Disease Control and Prevention criteria. Oral antibiotic prophylaxis (OAP) was considered as the administration of oral antibiotics the day before surgery combined with systemic intravenous antibiotic prophylaxis.

**Results:**

Of 3,701 patients, 2,518 (68%) underwent colon surgery and 1,183 (32%) rectal surgery. In colon surgery, the overall SSI rate was 16.4% and the organ-space SSI rate was 7.9%, while in rectal surgery the rates were 21.6% and 11.5% respectively (*p* < 0.001). Independent risk factors for organ-space SSI in colon surgery were male sex (Odds ratio -OR-: 1.57, 95% CI: 1.14–2.15) and ostomy creation (OR: 2.65, 95% CI: 1.8–3.92) while laparoscopy (OR: 0.5, 95% CI: 0.38–0.69) and OAP combined with intravenous antibiotic prophylaxis (OR: 0.7, 95% CI: 0.51–0.97) were protective factors. In rectal surgery, independent risk factors for organ-space SSI were male sex (OR: 2.11, 95% CI: 1.34–3.31) and longer surgery (OR: 1.49, 95% CI: 1.03–2.15), whereas OAP with intravenous antibiotic prophylaxis (OR: 0.49, 95% CI: 0.32–0.73) was a protective factor. Among patients with organ-space SSI, we found a significant difference in the overall 30-day mortality, being higher in colon surgery than in rectal surgery (11.5% *vs* 5.1%, *p* = 0.04).

**Conclusions:**

Organ-space SSI in colon and rectal surgery has some differences in terms of incidence, risk factors and outcomes. These differences could be considered for surveillance purposes and for the implementation of preventive strategies. Administration of OAP would be an important measure to reduce the OS-SSI rate in both colon and rectal surgeries.

## Background

Due to the clean-contaminated nature of the wound, rates of surgical site infections (SSI) after colorectal surgery are the highest among elective procedures, exceeding 20% in some institutions [1–3]. It has been suggested that the rates and risk factors for developing an SSI after colon and rectal surgery may be different [4, 5], due to the differences found in the surgical approach and the degree of bacterial contamination between both surgeries. Nevertheless, most studies carried out to date have analysed colon and rectal surgeries together [6, 7]. Separate assessments of patients undergoing colon and rectal surgery are scarce [4, 8].

It has been proposed that incisional SSI (I-SSI) and organ-space SSI (OS-SSI) may have distinct pathogenesis and risk factors. Incisional SSI has been associated with increased body mass index or the presence of an ostomy [6, 9]. On the other hand, OS-SSI has been more frequently related to blood transfusion, previous abdominal surgery or poor nutritional status [6, 7, 10]. Interestingly, the development of an OS-SSI has more severe consequences than the development of an I-SSI; in many cases OS-SSI requires reoperation and increases morbidity and length of stay (LOS) [11, 12]. Moreover, while many of the most significant advances in colon and rectal surgery such as laparoscopy and other minimally invasive techniques have decreased I-SSI rates, they have had a lesser impact on OS-SSI [13, 14].

Remarkably, the administration of mechanical bowel preparation (MBP) was discontinued in the last decades in most Spanish hospitals due to the lack of effectiveness [15]. In this scenario, and for reasons not well established, the administration of oral antibiotic prophylaxis (OAP) was discontinued too. Currently, only some hospitals use it in the elective surgery of the colon and rectum in Spain. This situation contrasts with that of other European and American countries, where the OAP is part of the daily practice.

The aim of this study was to compare the incidence, risk factors and outcomes of OS-SSI in patients undergoing elective surgery of the colon or rectum in a large, representative cohort of Spanish hospitals.

## Methods

### Patients, design and setting

We performed a multicentre observational study of a prospective cohort of adult patients (≥18 years old) undergoing elective colon and rectal surgery from January 2011 to December 2014 at 10 hospitals participating in the VINCat program. All consecutive patients hospitalized in any surgical department at the different hospitals were included and followed up until 30 days after surgery. Patients with a pre-existing SSI at the time of surgery were excluded. Post-discharge surveillance of SSI was mandatory and consisted of a review of electronic clinical records (primary and secondary care), checking readmissions and emergency visits, and reviewing microbiological and radiological data. For the purposes of the present study, patients were differentiated according to whether colon or rectal surgery was performed.

### VINCat surveillance program

The VINCat program [16] is a healthcare-associated infection surveillance program in Spain, based on the National Healthcare Safety Network (NHSN) model [17]. It recruits hospitals on a voluntary basis and currently receives surveillance data from trained infection control staff at 66 hospitals, who submit information on preoperative demographics, comorbidities, operative characteristics, microbiology and treatment data, and 30-day postoperative outcomes for eligible surgical procedures [18].

### Definitions

SSIs were defined according to the Centers for Disease Control and Prevention (CDC) criteria [19] and divided into superficial incisional, deep incisional and OS. Surgical procedure categories were stratified according to the risk of surgical infection (−1 to 3) as defined by the NHSN.

#### Independent variables

Predictor variables considered for the development of an OS-SSI were: age, sex, American Society of Anesthesiologists (ASA) physical status classification, MBP, OAP, adequacy of intravenous antibiotic prophylaxis, surgical risk index category according to the National Nosocomial Infections Surveillance (NNIS) modified system criteria [20], date and prolonged operation time (≥75^th^ percentile of the procedure), laparoscopy, wound classification, date of SSI, site of SSI (I-SSI or OS-SSI), microbiology and underlying disease (neoplasia, inflammatory bowel disease –IBD- or others). Age (<65 and ≥ 65 years), ASA (I-II and III-IV) score and NNIS modified risk index (−1-0 and 1–2) were dichotomized for the analysis.

Adequacy of intravenous antibiotic prophylaxis was established when all the following three factors were met: antibiotics administered according to local protocol at each hospital, completion of the infusion within 60 min before the surgical incision, and perioperative antibiotic redosing if indicated.

The OAP was always considered as the administration of oral antibiotic prophylaxis the day before surgery in combination with systemic intravenous antibiotic prophylaxis perioperatively. The administration was not mandatory and was done according to local protocols at each hospital. It was applied in 4 of the 10 participating hospitals.

#### Dependent variables

The development of overall SSI and OS-SSI in both colon and rectal populations, readmission, LOS and mortality within 30 days of initial surgery were recorded. Readmission for any cause within 30 days of initial surgery was documented. LOS included readmission if there was. Overall mortality was defined as death due to any cause within 30 days of initial surgery.

### Statistical analysis

Categorical variables were described as totals and frequencies; continuous variables were described as medians and interquartile ranges (IQR) and mean and standard deviation (SD) in some cases. Univariate analysis comparing the two populations was carried out using the chi-square test or Fisher exact test for categorical variables and the *t-*test or Mann-Whitney test for continuous variables. Comparisons between patients who developed an OS-SSI and those who did not (no OS-SSI) were performed separately for colon and rectal populations. Finally, multivariate analysis with all statistically significant variables (*p* ≤ 0.05) associated with OS-SSI in colon and rectal populations were performed separately to determine independent predictive factors for the development of OS-SSI. In these cases, results were given as odds ratios (OR) and 95% confidence intervals (95% CI). The final model’s goodness-of-fit was assessed by the Hosmer-Lemeshow test. Data were analysed with IBM SPSS 20.0 (Chicago, Ill.).

## Results

### Characteristics of patients and incidence of SSI in colon and rectal surgery

During the study period, a total of 3,701 patients undergoing elective colorectal surgery were prospectively followed-up, 68% after colon surgery and 32% after rectal surgery.

**Fig. 1:**
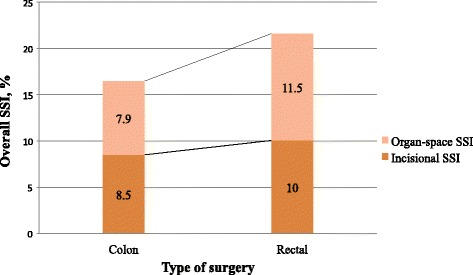
Incidence of surgical site infection in colon and rectal surgery. Shows the incidence of overall surgical site infection, incisional surgical site infection and organ-space surgical site infection in colon and rectal surgery separately. SSI: surgical site infection

Characteristics of patients undergoing colon or rectal surgery are shown in Table 1. Patients who underwent colon surgery were older (median age 70.6 years, interquartile range [IQR] 62–79 *vs* 68 years [IQR 60–76], *p* < 0.001) and had higher proportions of ASA score III-IV (42.2% *vs* 36.7%, *p* = 0.002) than patients undergoing rectal surgery. In contrast, patients undergoing rectal procedures were more likely to be male (67.2% *vs* 59.3%, *p* = 0.001), to have neoplasia (97% *vs* 93.5%, *p* < 0.001), to have a longer duration of surgery (42.7% *vs* 37.6%, *p* = 0.003), and to have an ostomy (64% *vs* 8.3%, *p* < 0.001). The administration of correct intravenous antibiotic prophylaxis was 84% in colon surgery and 81.6% in rectal surgery, *p* = 0.4. In colon surgery, the overall SSI rate was 16.4% and the OS-SSI rate 7.9%, while in rectal surgery, the overall SSI was 21.6% and the OS-SSI 11.5% (*p* < 0.001), as shown in Fig. 1. When patients who received OAP combined with correct intravenous antibiotic prophylaxis (*n* = 1.345) were analysed, significant differences in overall SSI rate between colon and rectal surgery (12.3% *vs* 19.9%, *p* < 0.001) were found, while there were no differences in the OS-SSI rate (6.2% vs 8.4%, *p* = 0.1).

**Table 1:** Characteristics of patients in colon and rectal surgery

Variable	Colon (*n* = 2518)	Rectum (*n* = 1183)	*p*-value
Age, median (IQR) years	70.6 (62–79)	68 (60–76)	<0.001
Age ≥65, *n* (%)	1711 (67.95%)	724 (61.20%)	0.001
Males, *n* (%)	1494 (59.33%)	795 (67.20%)	0.001
ASA III-IV, *n* (%)	1062 (42.18%)	434 (36.69%)	0.002
Neoplasia, *n* (%)	2355 (93.5%)	1147 (97%)	<0.001
Inflammatory bowel disease, *n* (%)	75 (3%)	16 (1.4%)	0.003
Other, *n* (%)	86 (3.4%)	17 (1.4%)	0.001
Duration of surgery ≥75th-percentile^a^, *n* (%)	947 (37.61%)	505 (42.69%)	0.003
NNIS Risk index 1–2, *n* (%)	909 (36.10%)	398 (33.64%)	0.15
Laparoscopy, *n* (%)	1515 (60.17%)	782 (66.10%)	0.001
Correct IV antibiotic prophylaxis, *n* (%)	2117 (84.07%)	966 (81.66%)	0.41
Previous radiotherapy, *n* (%)	33 (1.31%)	545 (46.07%)	<0.001
Previous chemotherapy, *n* (%)	78 (3.10%)	533 (45.05%)	<0.001
Oral antibiotic prophylaxis, *n* (%)	1078 (42.81%)	489 (41.34%)	0.41
Mechanical bowel preparation, *n* (%)	1749 (69.46%)	1038 (87.74%)	<0.001
- Missing	58 (2.30%)	20 (1.69%)	
Ostomy, *n* (%)	208 (8.26%)	754 (63.74%)	<0.001

*IQR* interquartile range, *ASA* American Society of Anesthesiologists physical status classification, *NNIS* National Nosocomial Infections Surveillance, *IV* intravenous

^a^Greater than 75th percentile for the duration of surgery (180 min, 3 h)

### Risk factors for OS-SSI in colon and rectal surgery

Univariate analyses of risk factors for OS-SSI in colon and rectal surgery are shown separately in Table 2. In colon surgery, male sex, NNIS ≥1 and ostomy creation were significantly associated with OS-SSI, while laparoscopic surgery and OAP had lower associations with OS-SSI. In rectal surgery, male sex, longer duration of surgery and NNIS ≥1 were associated with OS-SSI, whereas OAP had a lower association with OS-SSI.

**Table 2:** Univariate analysis of risk factors for organ-space surgical site infection in colon and rectal surgery

	Colon			Rectum		
Risk factor	No OS-SSI (*n* = 2318)	OS-SSI (*n* = 200)	*p*- value	No OS-SSI (*n* = 1043)	OS-SSI (*n* = 136)	*p*- value
Age, median (IQR) years	70 (61–79)	73 (63–79)	0.3	68 (60–76)	66.5 (58–74)	0.07
Age ≥ 65 years, (%)	67.6	72	0.2	61.5	58.8	0.5
Male sex, (%)	58.4	70	0.001	65.4	80.9	<0.001
ASA ≥ III, (%)	41.9	45.5	0.3	36.1	41.2	0.25
Correct IV antibiotic prophylaxis, (%)	84.3	81.5	0.3	81.4	83.8	0.5
Duration of operation ≥ p75th^a﻿^, (%)	37.5	39	0.7	41.2	54.4	0.003
Laparoscopy, (%)	61.6	44	<0.001	66.3	64.7	0.7
NNIS ≥ 1, (%)	35.5	43	0.03	32.7	41.2	0.05
Neoplasia, (%)	93.6	93	0.7	97.2	94.9	0.13
Inflammatory bowel disease, (%)	2.9	4	0.38	1.1	2.9	0.1
Chemotherapy, (%)	3.1	3.5	0.7	45.1	45.2	1
Radiotherapy, (%)	1.2	2.5	0.18	46	47.4	0.7
Oral antibiotic prophylaxis, (%)	43.7	33	0.004	43.3	26.5	<0.001
Mechanical bowel preparation, (%)	71.4	67.2	0.2	89.1	90.4	0.6
Ostomy, (%)	7.3	20	<0.001	63.8	65.2	0.7

*No OS-SSI* no organ-space surgical site infections (include patients with incisional SSI and patients without SSI), *OS-SSI* organ-space SSI, *IQR* interquartile range, *ASA* American Society of Anesthesiologists physical status classification, *IV* intravenous, *NNIS* National Nosocomial Infections Surveillance Risk Index.

^a^Greater than 75th percentile for the duration of surgery (180 min, 3 h)

A logistic regression multivariate analysis using significant predictive factors found in the univariate analysis is shown in Table 3. Independent risk factors for OS-SSI after colon surgery were male sex (OR 1.57, 95% CI 1.14–2.15) and ostomy creation (OR 2.65, 95% CI 1.8–3.9), while laparoscopy (OR 0.5, 95% CI 0.38–0.69) and the administration of OAP (OR 0.7, 95% CI 0.51–0.97) were independent protective factors. Independent risk factors for OS-SSI in rectal surgery were male sex (OR 2.11, 95% CI 1.34–3.31) and longer duration of surgery (OR 1.49, 95% CI 1–2.15), whereas the administration of OAP (OR 0.49, 95% CI 0.32–0.73) was the only independent protective factor.

**Table 3:** Multivariate analysis of risk factors for organ-space surgical site infection in colon and rectal surgery

	Colon				Rectum		
Risk factor	OR	95% CI	*p*-value	Risk factor	OR	95% CI	*p*-value
Male sex	**1.57**	**1.14–2.15**	**0.004**	Male sex	**2.11**	**1.34–3.31**	**0.001**
Laparoscopy	**0.5**	**0.38–0.69**	**<0.001**	Duration of operation ≥ p75th^a^	**1.49**	**1.03–2.15**	**0.07**
NNIS ≥ 1	1.17	0.83–1.64	0.36	NNIS ≥ 1	1.1	0.74–1.66	0.6
Oral antibiotic prophylaxis	**0.7**	**0.51–0.97**	**0.03**	Oral antibiotic prophylaxis	**0.49**	**0.32–0.73**	**0.001**
Ostomy	**2.65**	**1.8–3.92**	**<0.001**				

Signifficant OR and 95% CI appear in bold text

*OR* Odds ratio, *95%CI* 95% confidence interval, *NNIS* National Nosocomial Infections Surveillance Risk Index.

^a^Greater than 75th percentile for the duration of surgery (180 min, 3 h)

### Outcomes of patients with OS-SSI in colon and rectal surgery

Table 4 shows the outcomes of patients who developed an OS-SSI in colon and rectal surgery. There were no significant differences between colon and rectal procedures regarding median LOS (25 days [IQR 18–31] *vs* 23 days [IQR 16–33], *p* = 0.1), mean LOS (30.2 days ± SD 25 *vs* 32 days ± SD 28, *p* = 0.19) and readmission rate (19.5% *vs* 24.3%, *p* = 0.3). Overall 30-day mortality was significantly higher after colon surgery than after rectal surgery (11.5% [23/200] *vs* 5.1% [7/136], *p* = 0.04).

**Table 4:** Outcomes of patients with organ-space surgical site infection in colon and rectal surgery

Variable	Colon (*n* = 200)	Rectum (*n* = 136)	Overall (*n* = 336)	*p-*value
Readmission, *n* (%)	39 (19.5)	33 (24.3)	72 (21.4)	0.3
Readmission due to SSI, *n* (%)	34 (17)	30 (22.1)	64 (19)	0.2
Length of stay, median (IQR) days	25 (18–31)	23 (16–33)	24 (17–36)	0.1
Length of stay, mean (SD) days	30.2 (25)	32 (28)	27.6 (19.7)	0.1
Mortality, *n* (%)	23 (11.5)	7 (5.1)	30 (8.9)	0.04
Mortality attributed to SSI, *n* (%)	21 (10.5)	6 (4.4)	27 (8)	0.04

*SSI* surgical site infection, *﻿IQR* interquartile range, *SD* standard deviation

## Discussion

This large multicentre cohort study found significant differences in the incidence, predictive factors and outcomes of OS-SSI after elective colon and rectal surgery. This suggests that the two procedures should be considered as different surgical interventions.

The separation of procedures according to patients’ characteristics may allow more accurate assessment of their specific risk factors. Comparing colon and rectal populations, we found that they had different characteristics in terms of risk factors for SSI. Patients undergoing colon surgery were older, had more IBD and less laparoscopy, factors related to SSI. On the other hand, patients undergoing rectal surgery were younger but had more rate of malignancy; more frequently received chemoradiotherapy and had longer surgery duration. The surgical techniques were also different, something inherent to the anatomical location of the disease, in special with more ostomies performed in rectal resections. These factors, associated with the fact that the rectum has higher bacterial contamination load, conferred it greater risk of SSI. Accordingly, overall SSI and OS-SSI rates were higher in rectal surgery than in colon surgery. Although these rates were high, they were similar to these reported in previous studies [8, 21]. Data from surveillance systems in Europe an US vary widely [22, 23], being in most cases lower than ours, though post-discharge surveillance is not always performed.

We found significant differences in the predictive factors for developing an OS-SSI in colon and rectal surgeries. In colon surgery, independent risk factors predisposing to OS-SSI were male sex and ostomy creation, while laparoscopic surgery and OAP were protective factors. In rectal surgery, independent risk factors for OS-SSI were male sex and longer duration of surgery, whereas OAP was the only protective factor. Male sex was a common risk factor for developing OS-SSI in both colon and rectal surgeries; this association is well established [5, 7, 24], although the reasons are not known.

Ostomy creation was a strong risk factor for the development of OS-SSI in colon surgery but not in rectal surgery, as previously reported elsewhere [8]. Ostomies are normally used to divert the faecal stream from a newly created immature anastomosis, or to definitively disconnect the gastrointestinal tract in some extensive colorectal surgeries. Nevertheless, ostomies have been associated with increased rates of SSI in previous studies [4–6, 9] because they allow organisms from the air, contaminated hands, or skin flora to reach the subcutaneous fat and the wound, and eventually the intraabdominal cavity [25]. In our study, patients with colon surgery who received an ostomy more frequently underwent laparotomy due to complex pathology like IBD or diverticulitis. These diseases have been associated with OS-SSI [26], and ostomy creation may act, in part, as a marker of this complex pathology.

The laparoscopic approach significantly reduced SSI rates in several large-database studies and also offered other benefits such as faster recovery of pulmonary function, less pain and shorter postoperative stay [13, 14]. In our study it served as an independent protective factor for the development of OS-SSI in colon surgery, but not in rectal surgery. Probably, the beneficial effect of laparoscopy was exceeded by the higher frequency of risk factors for SSI inherent in rectal surgery.

Importantly, we found that OAP was a protective factor for the development of OS-SSI in both colon and rectal surgeries, although the impact was higher in rectal surgery, probably because the rectum has a higher level of bacterial contamination. During the study period there was not a national or regional recommendation for the application of OAP, and for this reason the use of the measure was decided by each participating hospital (it was only applied in 4 of the 10 hospitals). The findings of the present study lead to a change in the clinical practice of hospitals participating in the VINCat program and in 2016 the use of OAP was institutionally recommended. The OAP combined with intravenous prophylaxis and MBP significantly reduces SSI rates after colon and rectal surgery by decreasing the intraluminal bacterial load [27–30]; in a previous meta-analysis of randomized controlled trials comparing the effectiveness of OAP plus intravenous antibiotic prophylaxis *vs* intravenous antibiotic prophylaxis alone, the association of OAP was estimated to reduce the incidence of SSI by 43% [31]. Nevertheless, the use of MBP has been widely questioned, due to its unpleasant gastrointestinal effects, and in many studies it has failed to reduce SSI rates [15]. Currently, since almost all studies that demonstrate the effectiveness of OAP have been performed in combination with MBP, the use of MBP will have to be raised again. Last World Health Organization (WHO) recommendations on preoperative measures for surgical site infection prevention suggest using OAP with MBP in all adults undergoing elective colorectal surgery [32, 33].

Longer duration of surgery was an independent risk factor for the development of an OS-SSI in rectal surgery. This association has often been described in the colorectal surgery population [21, 34, 35], and it also favours other risk factors for SSI like the hyperglycaemia or hypothermia [33]. Given the capacity of this parameter to predict SSI, it was included as one of the components of the NNIS risk index. Rectal tumours close to the anal verge usually require extensive surgery with additional organ resection, requiring longer operative time and causing greater bleeding, factors that have been associated with an increased risk of SSI [24, 36]. Moreover, in these prolonged surgeries, antibiotic redosing is not always administered correctly.

Significantly, mortality of patients with OS-SSI after colon surgery was higher than after rectal surgery. The fact that patients in the colon group were older and more frequently had complicated diseases other than neoplasia could explain this result.

Among the strengths of the study is its multicentre nature, the large number of patients included and the fact that all data were collected by trained infection control staff. However, the study has a number of limitations that should be acknowledged. Firstly, the retrospective analysis of prospectively collected data may lead to bias and is unable to control for confounding factors. Secondly, certain risk factors that have been linked to SSI such as perioperative hyperglycaemia, hypothermia and blood transfusion were not recorded here.

## Conclusions

We found differences in the incidence, risk factors and outcomes of overall SSI and OS-SSI between colon and rectal surgery, suggesting that they could be considered as different surgical procedures. These differences should be borne in mind for the purpose of surveillance and for the implementation of preventive strategies. Administration of OAP would be an important measure to reduce the OS-SSI rate in both colon and rectal surgeries.

## Abbreviations

95% CI: 95% confidence interval
ASA: American Society of Anesthesiologists
CDC: Centers for disease control and prevention
IBD: Inflammatory bowel disease
IBM SPSS: International business machines Corp. Statistical package for the social sciences
IQR: Interquartile range
I-SSI: Incisional SSI
LOS: Length of stay
MBP: Mechanical bowel preparation
NHSN: National Healthcare Safety Network
NNIS: National Nosocomial Infection Surveillance
OAP: Oral antibiotic prophylaxis
OR: Odds ratio
OS-SSI: Organ-space surgical site infection
SD: Standard deviation
SSI: Surgical site infection
WHO: World Health Organization
